# Cryptotanshinone inhibits proliferation yet induces apoptosis by suppressing STAT3 signals in renal cell carcinoma

**DOI:** 10.18632/oncotarget.18483

**Published:** 2017-06-15

**Authors:** Zhiguo Chen, Rujian Zhu, Jiayi Zheng, Chen Chen, Chi Huang, Junjie Ma, Chen Xu, Wei Zhai, Junhua Zheng

**Affiliations:** ^1^ Department of Urology, Shanghai Tenth People's Hospital, Tongji University School of Medicine, Shanghai, China; ^2^ Department of Urology, The Affiliated Shanghai Tenth People's Hospital, Nanjing Medical University, Shanghai, China; ^3^ Department of Urology, Shanghai Pudong Hospital, Fudan University Pudong Medical Center, Shanghai, China; ^4^ Department of Pathology, Shanghai Tenth People's Hospital, Tongji University School of Medicine, Shanghai, China; ^5^ Department of Urology, Renji Hospital, Shanghai Jiao Tong University School of Medicine, Shanghai, China

**Keywords:** cryptotanshinone, renal cell carcinoma, cell proliferation, cell apoptosis, STAT3

## Abstract

It has been established that signal transducer and activator of transcription 3 serves as an oncoprotein in various human cancers; targeting it is therefore a reasonable approach for emerging cancer therapies. Cryptotanshinone, a natural compound extracted from the root of Salvia miltiorrhiza Bunge, has been identified as a potential STAT3 inhibitor. However, its functional role in renal cell carcinomas remains largely unknown. Therefore, we investigated the mode of action for cryptotanshinone. We found that cryptotanshinone substantially suppressed cancer cell growth while it promoted cell apoptosis by inhibiting the phosphorylation of STAT3 at Tyr705 and its blocking nuclear translocation. Coordinately, P-AKT, CyclinD1, C-MYC, MEKK2, and HGF were down-regulated and cell cycle progression was arrested at the G0/G1 phase, thereby attenuating cell proliferation. Moreover, the level of Cleaved-Caspase-3 was elevated while Bcl-2 and Survivin were down-regulated, accounting for the increased apoptosis. Furthermore, *in vivo* results revealed that cryptotanshinone effectively inhibits tumorigenesis in an A498-xenografted mouse model. Taken together, our data gives a more comprehensive understanding of how cryptotanshinone functions in renal cell carcinomas and demonstrates its potential as a powerful therapeutic approach to treat renal cell carcinomas.

## INTRODUCTION

Renal cell carcinoma (RCC) is among the ten most frequently occurring human cancers [1]. Approximately 30% of RCC patients have metastatic lesions that are detected during initial diagnosis [2]. Recently, targeted therapeutic agents, such as vascular endothelial growth factor (VEGF) receptor and mammalian target of rapamycin (mTOR) inhibitors have been frequently used in the treatment of RCC [3, 4]. Although the vast majority of patients show a remarkable clinical response, the therapeutic effects of these inhibitors are limited due to the development of a drug-resistant phenotype [5, 6]. Therefore, more potent and specific therapeutic strategies are urgently needed.

Signal transducer and activator of transcription 3 (STAT3) is a transcriptional factor and one of the most frequently activated STAT family members in human cancers. STAT3 plays an important role in controlling cell growth, survival, angiogenesis, and immune function [7]. Previous studies have identified STAT3 as a valid target for cancer therapy [8]. Notably, STAT3 is reported to be persistently activated in different types of RCCs and can serve as a prognostic marker [9, 10]. WP1066, a type of STAT3 inhibitor, exerts an anticancer effect on RCC cell lines and in a xenograft mouse model [11].

Salvia Miltiorrhiza Bunge is a classical herb with numerous demonstrated bioactivities, including anti-inflammatory, antioxidative stress, antiplatelet aggregation, and anticancer properties [12]. Cryptotanshinone (CPT) is a natural compound extracted from the root of Salvia miltiorrhiza Bunge that has displayed diverse anticancer properties against many human tumors, such as prostate cancer, leukemia, gliomas, lung carcinomas, hepatic carcinomas, pancreatic cancer, breast cancer, colorectal cancer, and melanoma cancer [13–22]. Mechanistically, CPT was identified as a potent STAT3 inhibitor [13, 15, 18, 20, 23]. However, the function of CPT in RCCs is yet to be elucidated. Therefore, we investigated the effect of CPT on RCC, and the underlying mechanisms by which it exerts these effects.

## RESULTS

### CPT inhibited RCC cell proliferation

**Figure 1:**
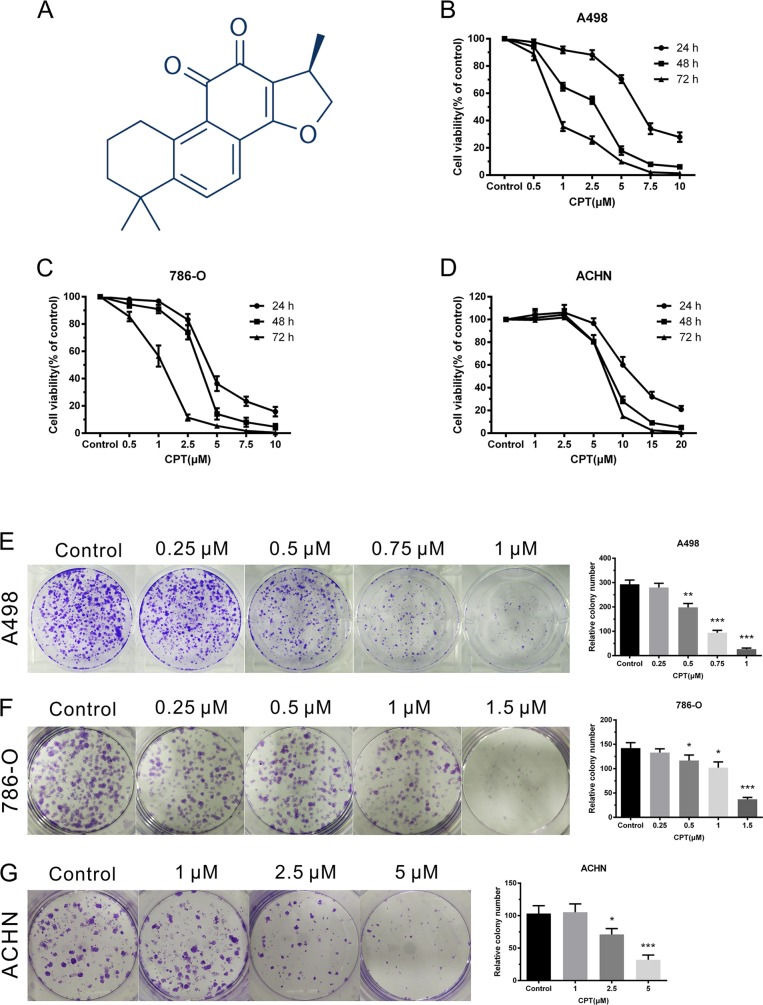
CPT inhibited RCC cell proliferation **(A)** The chemical structure of CPT. **(B) (C) (D)** CCK-8 assay of A498,786-O, and ACHN cells upon varying concentrations of CPT treatment for 24, 48, and 72 h. **(E) (F) (G)** Colony formation assays of A498,786-O, and ACHN cells with varying concentrations of CPT treatment for 48 h in 6-well plates. Representative images and average colony numbers are shown. Data are presented as the mean ± SD. *P < 0.05, **P < 0.01, ***P < 0.001 versus control group (n = 3).

The chemical structure of CPT is shown in Figure 1A. To assess the effect of CPT on cell proliferation, RCC cell lines were treated with varying concentrations of CPT for the indicated time. Cell proliferation was detected by CCK-8 and colony formation assays. The CCK-8 assay demonstrated that CPT inhibited the viability of various RCC cell lines (A498, 786-O, and ACHN) in both a time- and dose-dependent manner (Figure 1B-1D). Colony formation assays suggested that the colony forming ability of the A498, 786-O, and ACHN cells was remarkably reduced in a dose-dependent manner after treatment with CPT for 48 h (Figure 1E-1G). Comparably, the A498 and 786-O cells were more sensitive to CPT than the ACHN cells. These results indicate that CPT has anti-proliferative activity in RCC cells.

### CPT induced RCC cell cycle arrest

**Figure 2:**
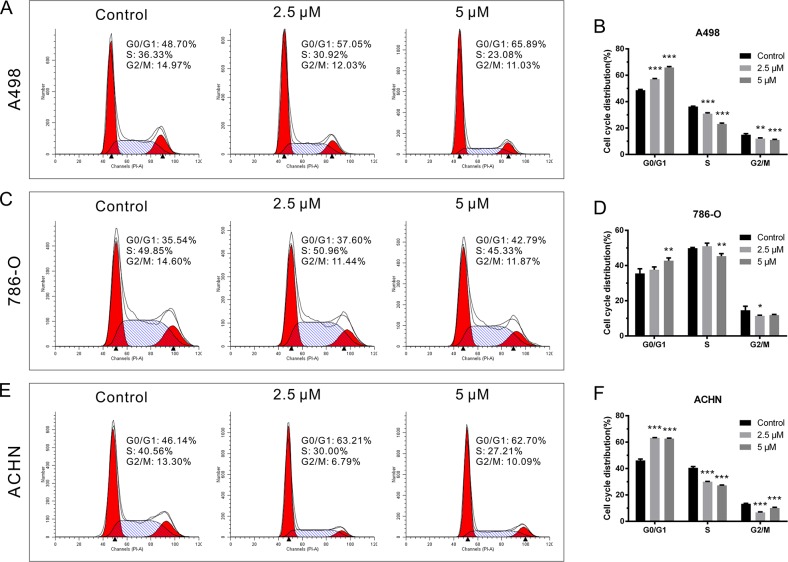
CPT induced RCC cell cycle arrest **(A) (C) (E)** The representative results of cell cycle analysis by flow cytometer assay in A498,786-O, and ACHN cells treated with different doses of CPT for 24 h (n = 3). **(B) (D) (F)** The G0/G1, S, and G2/M phase proportions of A498,786-O, and ACHN cells. Data are presented as the mean ± SD. *P < 0.05, **P < 0.01, ***P < 0.001 versus control group (n = 3).

To further investigate the potential mechanism by which CPT depressed RCC cell growth, cell cycle distribution was analyzed. RCC cell lines were treated with CPT (2.5 and 5 μM) for 24 h followed by flow cytometry assays. CPT caused G0/G1 phase arrest in A498 cells at a concentration of 2.5 μM (Figure 2A and 2B). Notably, the proportion of G0/G1 phase cells significantly increased as the concentration of CPT increased, which was accompanied by a decrease in the number of cells in S phase and G2/M phase (Figure 2A and 2B). The same concentration induced less dramatic results in 786-O cells (Figure 2C and 2D), while these trends were more noticeable in ACHN cells (Figure 2E and 2F). In general, with its impact on proliferation in RCC cell lines, CPT is likely associated with cell cycle arrest.

### CPT facilitated RCC cell apoptosis

**Figure 3:**
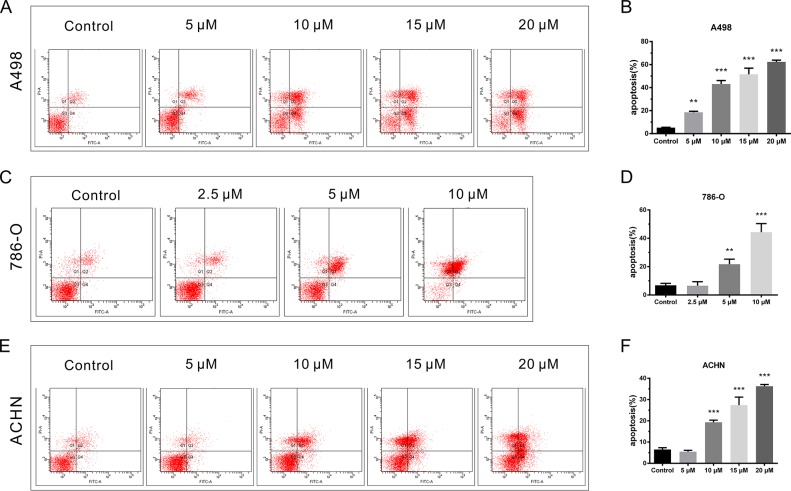
CPT facilitated RCC cell apoptosis **(A) (C) (E)** The representative results of cell apoptosis analysis by flow cytometer assay in A498,786-O, and ACHN cells treated with different doses of CPT for 48 h. **(B) (D) (F)** The percentages of surviving, early apoptotic, and late apoptotic cells of A498,786-O, and ACHN cells. Data are presented as the mean ± SD. *P < 0.05, **P < 0.01, ***P < 0.001 versus control group (n = 3).

Cell apoptosis was detected by Annexin V and PI staining. A498, 786-O, and ACHN cells were exposed to the indicated concentrations of CPT for 48 h. CPT significantly increased early apoptotic and late apoptotic cell populations, as well as decreased the viable cell population compared to vehicle control group, in a dose-dependent manner (Figure 3A-3F). The pro-apoptotic effect was most obvious in 786-O cells (Figure 3C and 3D). ACHN cells showed a higher tolerance of CPT at low concentrations compared with the other two cell lines (Figure 3E and 3F). Taken together, these results suggest that CPT pronouncedly induces apoptosis in RCC cells.

### CPT blocked P-STAT3 expression and translocation

**Figure 4:**
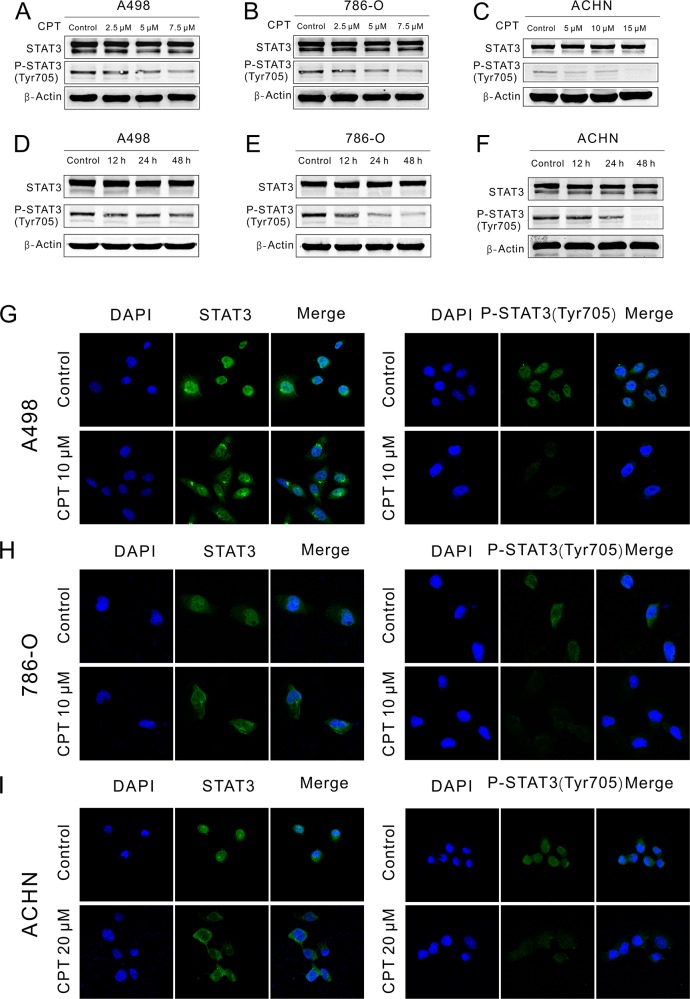
CPT blocked P-STAT3 expression and translocation **(A) (B) (C)** A498,786-O, and ACHN cells were plated in 6-well plates and treated with varying concentrations of CPT for 48 h. STAT3 and P-STAT3 (Tyr705) expression was detected by Western blotting. β-Actin was used as a loading control. **(D) (E) (F)** A498,786-O, and ACHN cells were plated in 6-well plates and treated with CPT for 12, 24 h, and 48 h. STAT3 and P-STAT3 (Tyr705) expression was detected by Western blotting. β-Actin was used as a loading control. **(G) (H) (I)** A498, 786-O and ACHN cells were treated with CPT for 2 h. STAT3 and P-STAT3(Tyr705) location was detected by immunofluorescence staining assay. Representative immunofluorescence analyses by a confocal microscopy are shown. The nuclei were stained with DAPI. Scale bar =10 μm. Three different independent experiments were performed.

To assess the effect of CPT on STAT3 activation and nuclear translocation, Western blot and immunofluorescence staining assays were performed, respectively. After treatment with different concentrations of CPT for different lengths of time, the protein level of STAT3 and P-STAT3 (Tyr705) was detected by Western blot in RCC cell lines. CPT obviously abolished the phosphorylation of STAT3 at Tyr705 without effecting the level of total STAT3 in A498, 786-O, and ACHN cells in a dose- (Figure 4A-4C) and time-dependent manner (Figure 4D-4F). Furthermore, after treatment with CPT for 2 h, STAT3 translocation was detected by immunofluorescence staining in RCC cell lines. As shown in Figure 4G-4I, the nuclear translocation of STAT3 and P-STAT3 (Tyr705) was attenuated, while the fluorescence intensity of P-STAT3 (Tyr705) was also blunted. These data demonstrate that CPT minimizes the phosphorylation of STAT3 at Tyr705 and thereby its nuclear translocation in RCC cell lines.

### CPT repressed P-AKT/C-MYC signaling while enhanced Caspase-3/Bcl-2 signaling

**Figure 5:**
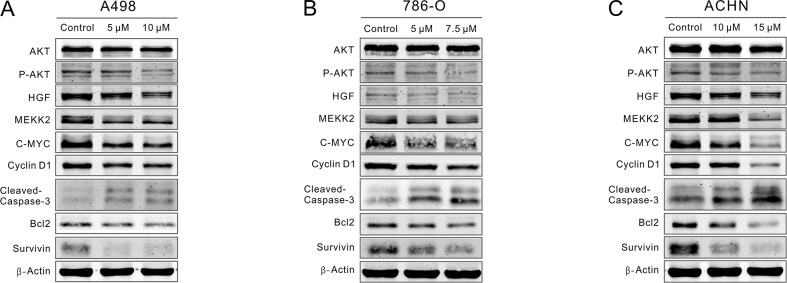
CPT repressed P-AKT/C-MYC signaling while enhanced Caspase-3/Bcl-2 signaling. A498,786-O, and ACHN cells were treated with varying concentrations of CPT for 48 h **(A) (B) (C)** The expression levels of critical cell cycle regulators, proliferation-related proteins, and apoptosis-related proteins that detected by Western blot. β-Actin was used as a loading control. Three independent experiments were performed.

As activated STAT3 modulates the expression of proteins that involved in cell cycle, proliferation and apoptosis, we investigated whether CPT could control some critical STAT3 signaling-associated proteins. RCC cells were treated with CPT for 48 h, and we detected the expression of AKT, P-AKT, C-MYC, HGF, MEKK2, Cyclin D1, Bcl-2, Survivin, and Cleaved-Caspase-3 by Western blot. We found that the expression of P-AKT, C-MYC, HGF, MEKK2, and Cyclin D1 was significantly suppressed by CPT (Figure 5). Therefore, CPT reduced P-AKT/C-Myc signaling. In addition, the up-regulation of Cleaved-Caspase3 and down-regulation of anti-apoptosis proteins (Bcl-2 and Survivin) indicated the activation of the Caspase-3/Bcl-2 signaling pathway, which was also in agreement with the results of the flow cytometry assays (Figure 5). In conclusion, CPT reduced P-AKT/C-MYC signaling while it enhanced Caspase-3/Bcl-2 signaling in RCC cell lines.

### CPT attenuated cell growth while activated cell apoptosis *in vivo*

**Figure 6:**
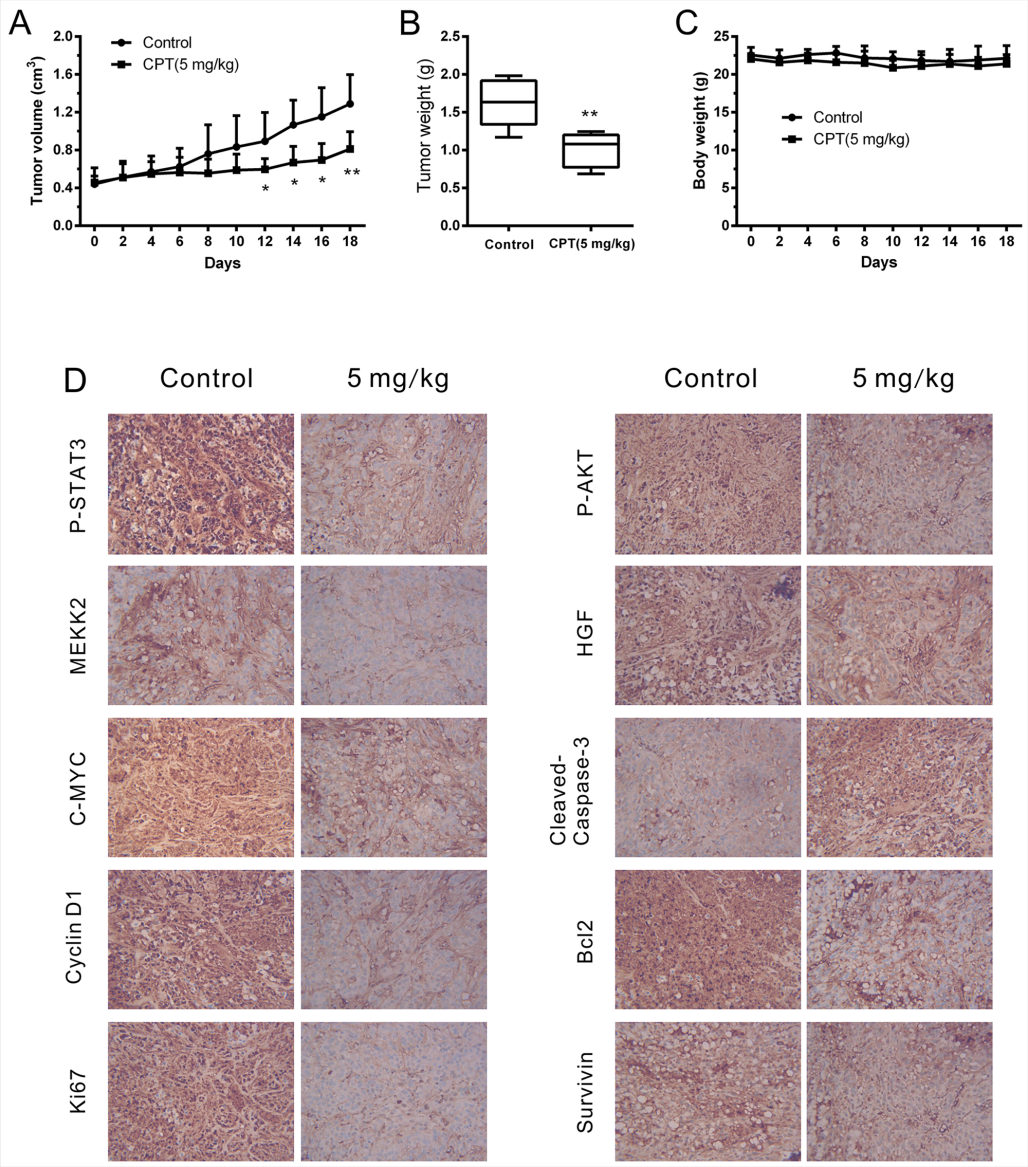
CPT attenuated cell growth while activated cell apoptosis *in vivo*. A498-xenografted nude mice (n = 6 per group) were injected with vehicle or CPT (5 mg/kg) for 18 days **(A)** Statistical analysis of tumor volume. **(B)** Statistical analysis of tumor weight. **(C)** Statistical analysis of body weight. **(D)** Immunohistochemical staining results of indicated proteins in xenograft tumor tissues. The sections were examined by light microscopy under × 400 magnification. The data were presented as the mean ± SD. *P < 0.05, **P < 0.01 versus control group.

Furthermore, an RCC xenograft mouse model was established to detect the anticancer activities of CPT *in vivo*. The mice were randomly divided into vehicle or CPT treatment groups when the tumors grew to approximately 300 mm^3^ (6 mice/group). Then, they were treated with vehicle or CPT (5 mg/kg) for 18 days. We found that CPT effectively reduced tumor volume (Figure 6A) and tumor weight (Figure 6B) without affecting body weight (Figure 6C). Significant differences in tumor volume were detected around the 12^th^ treatment day (P < 0.05). To investigate any changes in the levels of proteins identified *in vitro*, tumor tissues were used for IHC staining. As shown in Figure 6D, P-STAT3 (Tyr705) was significantly suppressed in tumors from mice treated with CPT. Moreover, the expression of P-AKT, C-MYC, HGF, MEKK2, Cyclin D1, Bcl-2, Survivin, and Ki67 decreased while Cleaved-Caspase3 expression increased in the CPT treatment group. All these results were consistent with *in vitro* observations, indicating that CPT can attenuate cell growth while activating cell apoptosis *in vivo*.

**Figure 7:**
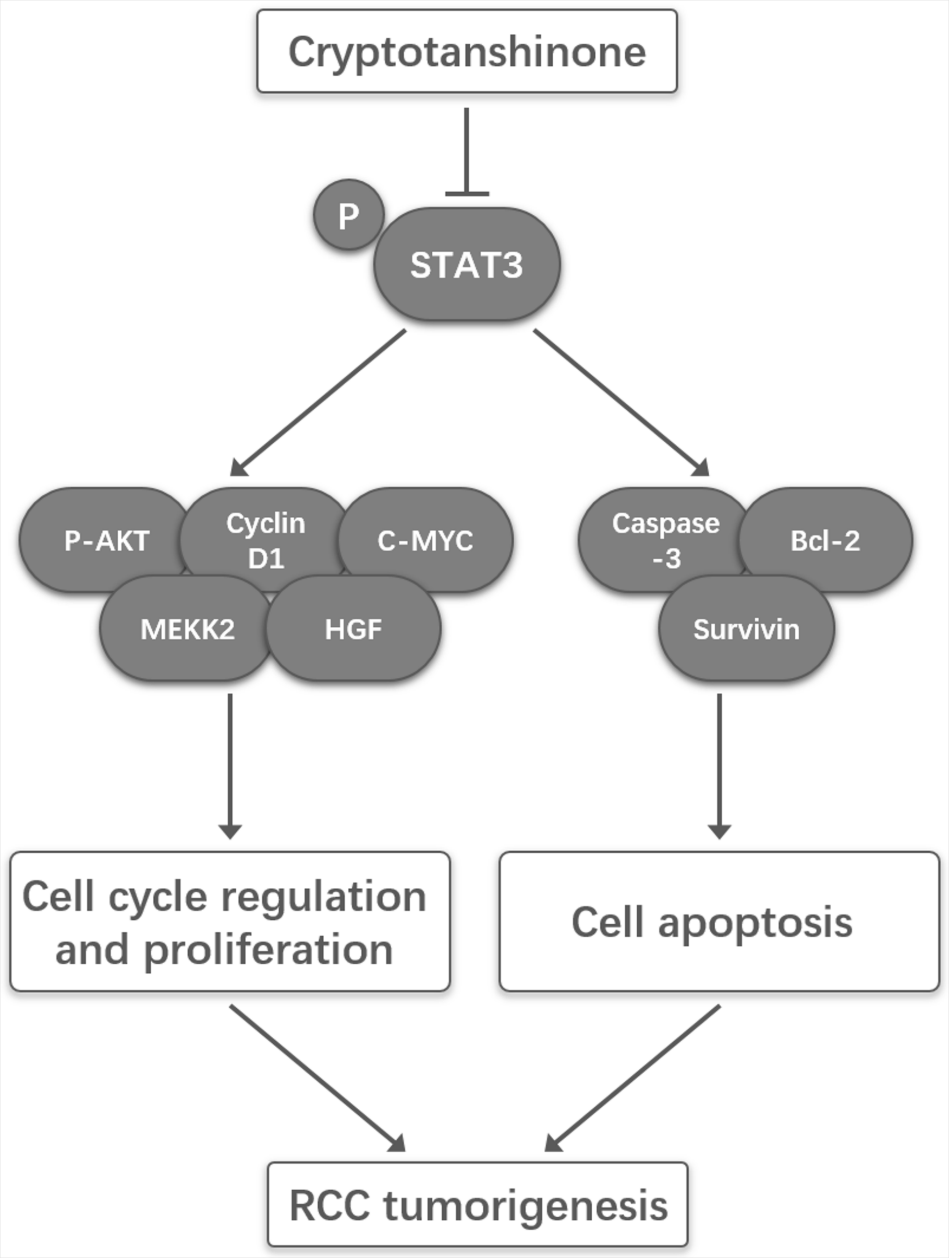
A schematic illustration of the proposed model depicting that CPT inhibited proliferation yet induced apoptosis by suppressing STAT3 signals in renal cell carcinoma

A schema summarizing the underlying mechanisms that CPT inhibited proliferation yet induced apoptosis in RCC was provided in Figure 7.

## DISCUSSION

The anticancer activity of CPT has been investigated in many human cancers. It can suppress cell proliferation, induce cell apoptosis, impair cell migration and invasion, and induce autophagic cell death [17, 21, 22]. The potential previously unexplored mechanisms included suppressing STAT3 signals [18], enhancing tumor necrosis factor alpha (TNF-α)-induced apoptosis [14], activating the AMP-activated protein kinase signaling pathway [17], suppressing estrogen receptor signaling [19], inhibiting NF-kB signaling [21], down-regulating androgen receptor signaling [24, 25], augmenting Fas-induced apoptosis [26], or inducing endoplasmic reticulum stress-mediated apoptosis [27, 28]. Previous studies have shown that STAT3 is persistently activated in RCC specimens [9, 10], and STAT3 inhibition can block proliferation and induce apoptosis in RCC cell lines or a xenograft mouse model [29, 30]. Therefore, we investigated the effects of CPT (a STAT3 inhibitor) on human RCC cell lines and in a xenograft mouse model.

In this study, we confirmed that CPT markedly suppressed the phosphorylation of STAT3 at Tyr705 and attenuated nuclear translocation *in vitro* and *in vivo*, consistent with previous studies [20]. However, these studies did not report whether CPT could abrogate STAT3 translocation using cell lines. Similarly, another study used DU145 prostate cancer cells to show that CPT inhibits constitutive STAT3 functions by blocking dimerization, but there was lack evidence in a xenograft mouse model [13].

Here, we demonstrated that cell cycle progression was arrested at the G0/G1 phase using flow cytometry assays; inhibition occurred through the down-regulation of cell growth related proteins including P-AKT, CyclinD1, C-MYC, MEKK2, and HGF. However, several studies reported that after treatment with CPT, G2/M phase arrest was increased, which was accompanied by a slight S phase arrest in acute lymphoblastic leukemia cells [21]. Moreover, CPT arrested the cell cycle at S phase in PC-3 cells via the suppression of cdc2 [31]. In LNCaP cells, a low concentration of CPT induced G1 arrest, while a higher concentration resulted in G2/M arrest [25]. In conclusion, these results suggest that the inhibitory effect of CPT on cell cycle arrest might be different depending on the cell lines and drug concentrations used.

Cell apoptosis increased following activation of the Caspase-3/Bcl-2 signaling pathway. Similarly, with the up-regulation of cleaved-caspases-3 and down-regulation of Bcl-2, CPT induced apoptosis in melanoma cells and colorectal cancer cell lines [20, 22]. In addition, acute lymphoblastic leukemia cell apoptosis increased due to the loss of mitochondrial membrane potential and up-regulation of cleaved Caspase 3/7, Caspase 9, and poly ADP ribose polymerase (PARP) [21]. These results revealed that CPT might induce apoptosis through the mitochondria-mediated apoptotic pathway. Moreover, another study found that CPT could sensitize TNF-α-induced apoptosis through ROS-dependent activation of Caspase-8 and p38 in human myeloid leukemia KBM-5 cells [14]. Another study indicated that CPT could significantly block activation of JNK and p38 MAPK, which suppressed the expression of Bcl-2 and eventually sensitized DU145 prostate cancer cells to Fas (APO1/CD95)-mediated apoptosis [26]. Another study from this group demonstrated that CPT served as a potent stimulator of endoplasmic reticulum stress and induced apoptosis in HepG2 and MCF7 cells [27]. Interestingly, CPT also induced autophagic cell death in HepG2 cells and colon cancer cells [17, 32]. Taken together, in addition to the mitochondrial apoptosis pathway, other potential mechanisms were involved in the pro-apoptotic effects of CPT.

Although we focused only on the effect of inhibiting proliferation and inducing apoptosis, CPT also played an anti-metastasis effect in tumorigenesis [22]. Whether CPT can minimize invasion and metastasis in RCC remains unclear and requires further investigation. Recent studies indicated that P-STAT3 was increased in sunitinib-resistant RCC xenografts [33]. Interestingly, our study showed that CPT pronouncedly attenuated the expression of P-STAT3. Whether CPT can reverse sunitinib-resistance and alter the efficacy of sunitinib therapy for RCCs remains to be determined.

In summary, our study demonstrated that CPT has anticancer activity in RCCs as an STAT3 inhibitor, which could be developed as a potential therapeutic agent for RCC.

## MATERIALS AND METHODS

### Antibodies and reagents

Antibodies against STAT3, P-STAT3 (Tyr705), AKT, P-AKT, C-MYC, Cyclin D1, Cleaved-Caspase-3, HGF, MEKK2, Survivin, and Bcl-2 were purchased from Abcam (Cambridge, MA, USA). CPT was purchased from Selleckchem (Houston, TX, USA), and dissolved in dimethyl sulfoxide (DMSO; 10 mM) for subsequent experiments.

### Cell culture

Dulbecco's modified Eagle's medium (DMEM), Minimum Essential Medium (MEM), RPMI-1640, 0.25% Trypsin–EDTA, and Fetal Bovine Serum (FBS) were purchased from Gibco (Life Technologies, Grand Island, NY, USA). The human RCC A498, 786-O, and ACHN cell lines were purchased from the American Type Culture Collection (ATCC, Manassas, VA) and cultured at 37°C with 5% CO_2_. A498 cells were cultured in DMEM. ACHN cells were cultured in MEM. 786-O cells were cultured in DMEM. All the media was supplemented with 10% FBS and 1% penicillin-streptomycin.

### Cell Counting Kit-8 (CCK-8) assays

Cell proliferation was assessed using the Cell Counting Kit-8 (CCK-8) (Dojindo, Kumamoto, Japan). Briefly, RCC cells were seeded into 96-well plates (3×10^3^ cells/well) and incubated overnight for attachment. Then, they were treated with DMSO or varying concentrations of CPT in complete medium (200 μL) for the indicated time. There were 5 replicate wells for each concentration. After incubation for 24, 48, or 72 h, the medium was replaced with normal medium (100 μL). Then, 10 μL of CCK-8 solution was added to each well and incubated at 37°C incubator with 5% CO_2_ for 2 h. The absorbance at 450 nm was measured by a microplate spectrophotometer (BioTek, USA).

### Colony formation assays

RCC cells were seeded into 6-well plates and allowed to grow overnight. Cells were then exposed to DMSO or different concentrations of CPT. After being cultured for 48 h, the medium was replaced with normal medium. Eight days later, the cells were washed with Phosphate Buffered Saline (PBS) gently, and then the colonies were fixed with 95% ethanol and stained with 0.1% crystal violet. The number of colonies (> 50 cells) in each group was counted under a microscope.

### Cell cycle analysis

PI/RNase Staining Buffer (BD Biosciences, San Jose, CA, USA) was used to analyze the cell cycle. RCC cells were harvested during the logarithmic phase of growth and seeded into 6 cm tissue culture dishes. After treatment with DMSO or different concentrations of CPT for 24 h, the cells were harvested with EDTA-free trypsin, washed with ice-cold PBS, and fixed with 75% alcohol at 4°C overnight. Then, the cells were resuspended in 0.2 mL PI/RNase Staining Buffer in a tube. After incubating for 30 min in the dark at 4°C, cells were analyzed using a BD FACSCanto II flow cytometer (Becton Dickinson, Franklin Lakes, NJ, USA). The cell cycle distribution was analyzed with ModFit LT software (Verity Software House, Topsham, ME).

### Cell apoptosis analysis

A FITC Annexin V Apoptosis Detection Kit (BD Biosciences, San Jose, CA, USA) was used to detect apoptotic cells. RCC cells were seeded into 6 cm tissue culture dishes and treated with DMSO or varying concentrations of CPT. After culturing for 48 h, all the cells were collected in a tube and resuspended in 50 μL binding buffer together with 2.5 μL Annexin V-FITC. The tubes were incubated for 15 min at room temperature in the dark. Then, 250 μL binding buffer containing 5 μL PI was added to each tube. All the samples were immediately analyzed using a BD FACSCanto II flow cytometer (Becton Dickinson, Franklin Lakes, NJ, USA).

### Immunofluorescence staining

After treating with CPT or vehicle (DMSO) for only 2 h, cells were washed with PBS and fixed with 4% paraformaldehyde for 15 min at room temperature, followed by permeabilization with 0.2% Triton X-100 in PBS for 10 min. Non-specific antigenic sites were blocked with 1.0% bovine serum albumin (BSA) in PBS for 1 h. Subsequently, the cells were incubated with P-STAT3(Tyr705) or STAT3 antibodies (diluted 1:200 in blocking buffer) overnight at 4°C. After washing with PBS, cells were incubated with AlexaFluor 488-labeled secondary antibody (diluted 1:1000 in blocking buffer) for 1 h in the dark and then washed with PBS. Coverslips were mounted in Prolong Gold antifade reagent with DAPI (Molecular Probes, Eugene, OR, USA). Cells were observed with a confocal microscope (Zeiss, Jena, Thuringia, Germany).

### Western blotting

RCC cells were seeded into 6-well plates and treated with CPT or DMSO (vehicle control) for the indicated time. Then, the cells were collected and lysed in RIPA lysis buffer (150 mM NaCl, 50 mM Tris pH 7.4, 1% TritonX-100, 1% sodium deoxycholate, 0.1% SDS, and protease inhibitors). The protein concentration was measured using a BCA Protein Assay Kit (Beyotime Institute of Biotechnology, Jiangsu, China). Fifty μg protein was separated by 10% sodium dodecyl sulfate polyacrylamide gel electrophoresis (SDS-PAGE) and transferred onto nitrocellulose membranes using a wet transfer apparatus (Bio-Rad, Hercules, CA, USA). Membranes were blocked with 3% Bovine Serum Albumin (BSA) in PBST buffer (PBS containing 0.1% Tween-20) at room temperature for 2 h and incubated with primary antibodies overnight at 4°C. After washing with PBST three times, membranes were incubated with the corresponding secondary antibodies for 2 h at room temperature. Protein bands were detected with the Odyssey scanner (LI-COR Biosciences, USA) after three washes with PBST.

### Xenograft studies

Five-week-old male athymic nude mice were used for this experiment. A498 cells were resuspended in a mixture of PBS and matrigel (BD BioSciences, San Jose, CA). Cells (2 × 10^6^) were injected subcutaneously into the right flank of nude mice. When the tumors grew to approximately 300 mm^3^, the mice were randomly divided into vehicle or CPT treatment groups (6 mice/group). Then, they were treated with vehicle (10% DMSO + 30% PEG 300 + 5% Tween 80 + ddH_2_O) or 5 mg/kg CPT that was dissolved in the vehicle via intraperitoneal injection (every other day for 18 days). Body weight and tumor volume were recorded every two days. Tumor length (L) and width (W) were measured with a vernier caliper, and the tumor volume (TV) was calculated by the following formula: TV = (L× W^2^)/2. All mice were sacrificed at day 20, and the tumors were collected.

### Immunohistochemical staining in xenograft tumors

All the xenograft tumors were fixed in 4% paraformaldehyde and then sliced into paraffin sections for IHC assays. The sections were deparaffinized, and endogenous peroxidase was destroyed with 3% H_2_O_2_. Non-specific antigenic sites were blocked with BSA in PBS for 30 min at room temperature. Subsequently, the tissues were incubated with primary antibodies overnight at 4°C, followed by incubation with goat anti-rabbit IgG antibodies. The immunoreactivities were visualized using DAB. Finally, the tissues were counterstained with hematoxylin, mounted, and observed under a microscope.

### Statistical analysis

All the results were obtained from at least three independent experiments. Data are presented as the mean ± standard deviation (SD). The Student's t-test and one-way ANOVA were used to make a statistical comparison between groups with SPSS 20.0 software. P < 0.05 was considered statistically significant.

## Abbreviations

CPT: cryptotanshinone
RCC: renal cell carcinoma
STAT3: signal transducer and activator of transcription 3
DMSO: dimethyl sulfoxide
